# A rare presentation of atypical demyelination: tumefactive multiple sclerosis causing Gerstmann’s syndrome

**DOI:** 10.1186/1471-2377-14-68

**Published:** 2014-04-02

**Authors:** Sharmilee Gnanapavan, Zane Jaunmuktane, Kelly Pegoretti Baruteau, Sakthivel Gnanasambandam, Klaus Schmierer

**Affiliations:** 1 Blizard Institute, Barts and The London School of Medicine & Dentistry, Queen Mary, University of London, London, UK; 2 Department of Neurology, Barts Health NHS Trust, The Royal London Hospital, London, UK; 3 Division of Neuropathology, UCLH Foundation Trust, The National Hospital for Neurology & Neurosurgery, Queen Square, London, UK; 4 Department of Neuroradiology, Barts Health NHS Trust, The Royal London Hospital, London, UK

**Keywords:** Gerstmann’s syndrome, Tumefactive MS, Demyelination, Aphasia, Agnosia, Left-right disorientation, Dyscalculia, Natalizumab

## Abstract

**Background:**

Tumefactive demyelinating lesions are a rare manifestation of multiple sclerosis (MS). Differential diagnosis of such space occupying lesions may not be straightforward and sometimes necessitate brain biopsy. Impaired cognition is the second most common clinical manifestation of tumefactive MS; however complex cognitive syndromes are unusual.

**Case presentation:**

We report the case of a 30 year old woman who presented with Gerstmann’s syndrome. MRI revealed a large heterogeneous contrast enhancing lesion in the left cerebral hemisphere. Intravenous corticosteroids did not stop disease progression. A tumour or cerebral lymphoma was suspected, however brain biopsy confirmed inflammatory demyelination. Following diagnosis of tumefactive MS treatment with natalizumab effectively suppressed disease activity.

**Conclusions:**

The case highlights the need for clinicians, radiologists and surgeons to appreciate the heterogeneous presentation of tumefactive MS. Early brain biopsy facilitates rapid diagnosis and management. Treatment with natalizumab may be useful in cases of tumefactive demyelination where additional evidence supports a diagnosis of relapsing MS.

## Background

Gerstmann’s syndrome is a rare disorder resulting from damage to the angular gyrus of the dominant parietal lobe leading to dysgraphia, dyscalculia, finger agnosia and left-right disorientation [1]. In adults this syndrome is usually seen after stroke. In younger patients inflammation, malignancy and abscesses need to be considered [2].

## Case presentation

A 30 year old, right-handed woman was admitted with a two week history of word finding difficulties. There was subtle expressive and receptive dysphasia alongside acalculia, agraphia, finger agnosia and left-right disorientation (Additional file 1), and right-sided homonymous hemianopia.

**Figure 1:**
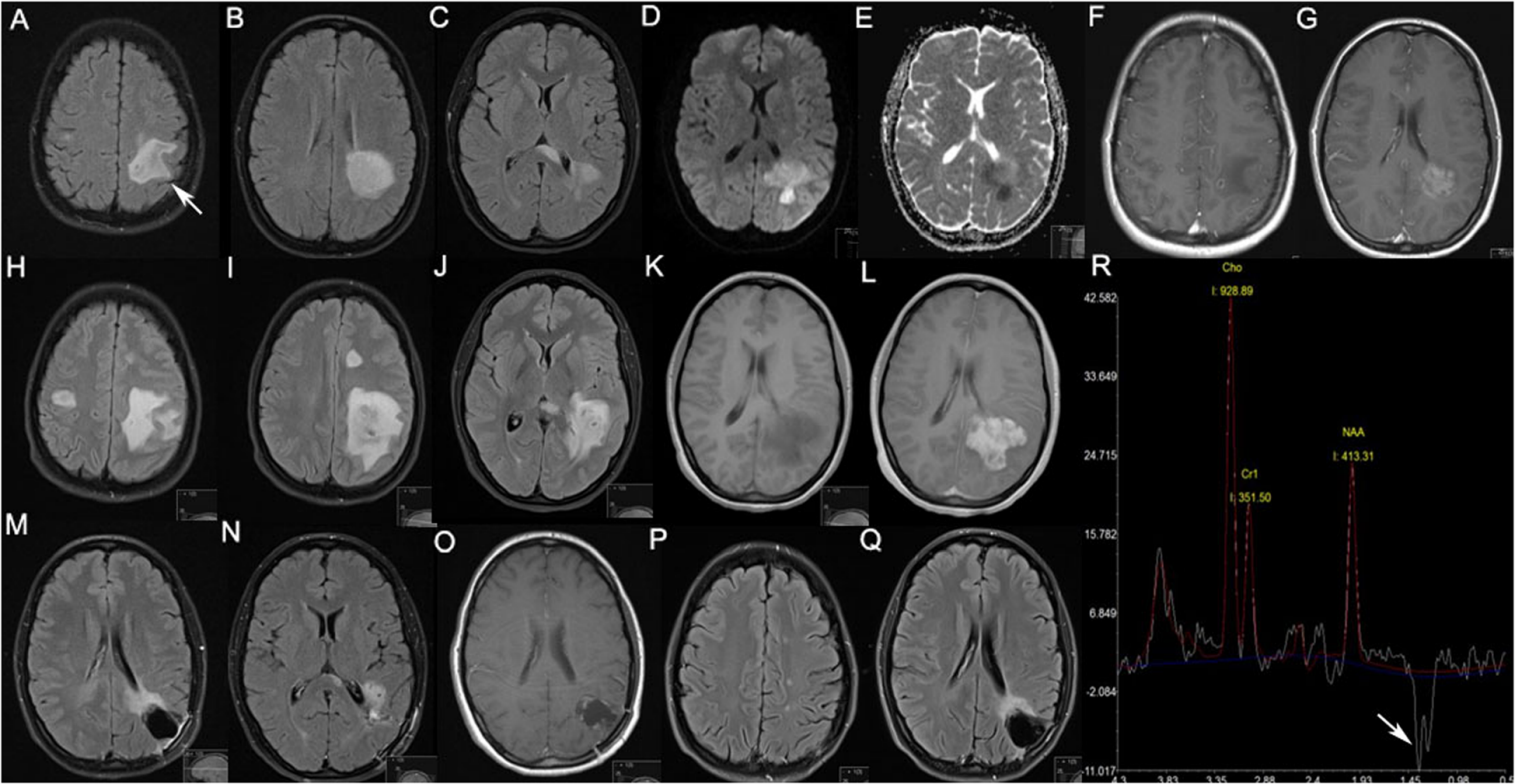
**Axial MRI and MR spectroscopy data acquired at 3 Tesla in a 30 year old woman with tumefactive MS.** Techniques used were fluid-attenuated inversion recovery (FLAIR; **A**-**C**, **H**-**J**, **P**, **Q**), diffusion weighted imaging **(DWI; D)** and apparent diffusion coefficient **(ADC; E)** maps, T_1_ before **(F, K)** and after administration of gadolinium contrast **(G, L, O)**, respectively. At presentation **(A-G)**, FLAIR showed a large area of high signal in the left occipito-temporal region including the angular gyrus (arrow in **A**) and extending to the genu of the corpus callosum **(A-C)**, while DWI and ADC maps showed partially restricted diffusion **(D, E)**. Significant enhancement occurs after injection of gadolinium **(G)**. Two weeks after presentation **(H-L)** FLAIR **(H-J)** showed increase in size of the index lesion, and three additional lesions, two in the frontal lobes **(H, I)** and one in the left occipital lobe (data not shown). All four lesions enhanced after application of gadolinium (only index lesion shown in **L**). Following brain biopsy, and four weeks after the first natalizumab infusion **(M-O)**, there is significant reduction in size of the index lesion with evidence of post-surgical cavity and minimal residual gadolinium enhancement **(O)** Fourteen months after first presentation, and after 13 courses of natalizumab, no evidence of disease activity was detected **(P, Q)**. Long echo MR spectroscopy of the tumefactive lesion at presentation revealed significantly reduced NAA/Cr ratio, increased Cho/Cr ratio and an inverted lactate doublet curve (arrow) **(R)**.

Magnetic resonance imaging (MRI) of the head demonstrated a large heterogeneous lesion associated with focal cystic changes in the left occipito-temporal lobes with partial restriction on diffusion weighted MRI (DWI) and heterogeneous gadolinium enhancement (Figure 1A-G). No spinal cord lesions were detected. Long echo time proton MR spectroscopy (MRS) of the lesion revealed an n-acetyl aspartate (NAA)/creatine (Cr) ratio of 1.17, a choline (Cho)/Cr ratio of 2.64 and an inverted lactate doublet curve (Figure 1R). Cerebrospinal fluid (CSF) analysis showed normal cell count (white cells <1, red cells <1), protein of 0.39 g/L, IgG of 21 mg/L, negative microbiology screen, however oligo-clonal bands predominantly in CSF compared to serum.

She was treated with intravenous (IV) methylprednisolone (MP), 1 g daily for three consecutive days with no clinical effect. Two weeks later the patient developed severe headache with intractable vomiting. Repeat MRI head revealed increase in size of the previously detected lesion and three new lesions (Figure 1H-L).

**Figure 2:**
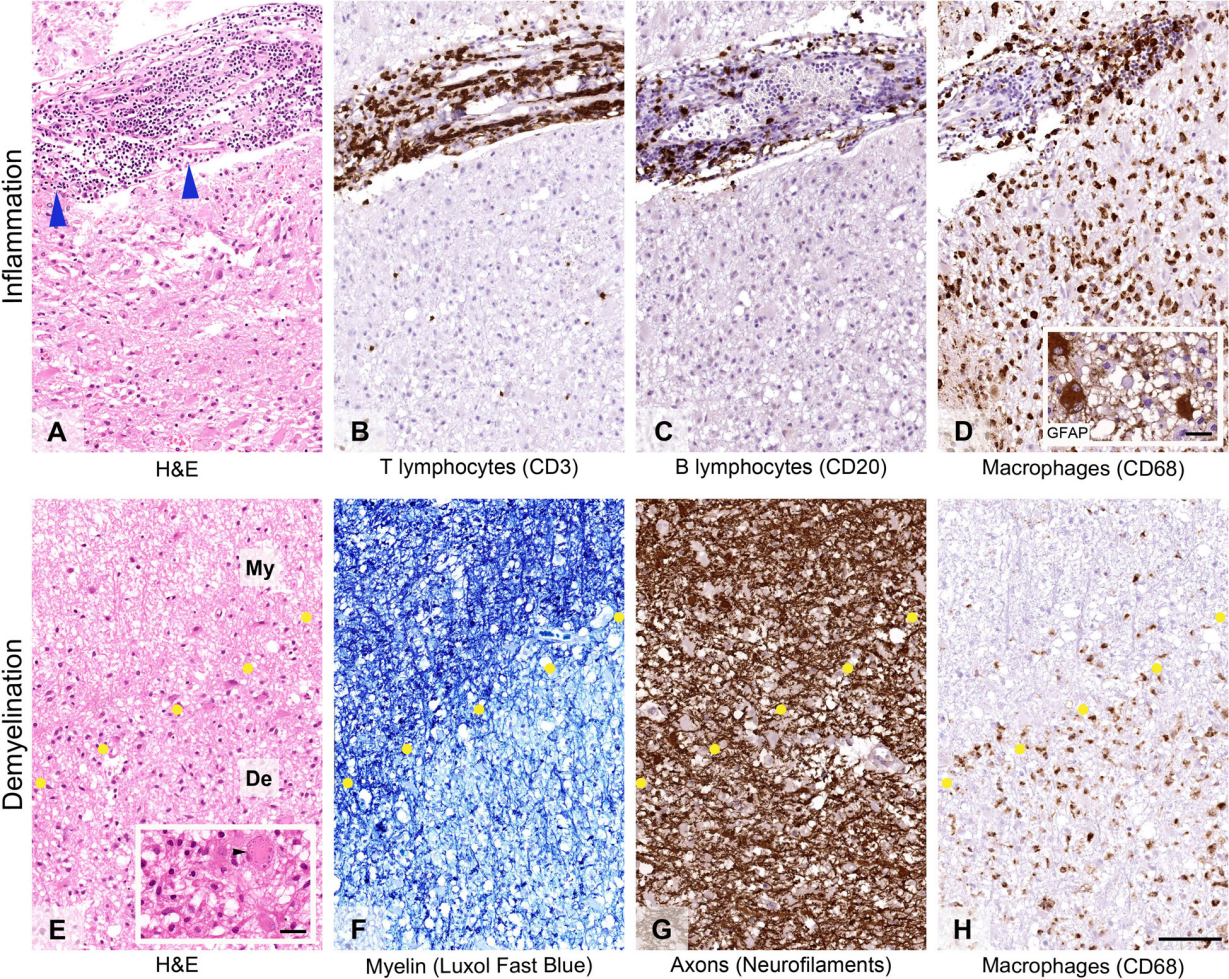
**Brain biopsy of the tumefactive multiple sclerosis lesion.** Inflammation **(A-D)**: Haematoxylin-Eosin **(H & E)** stained section **(A)** reveals perivascular infiltrate of mononuclear inflammatory cells (blue arrowheads). Many of the inflammatory cells are CD3 positive T cells **(B)** some of which show spilling in the surrounding neural parenchyma. Fewer perivascular lymphocytes are CD20 positive B cells **(C)**. There are also frequent macrophages around the blood vessels and diffusely in the neural parenchyma **(D)**. The macrophages are intermingled with glial fibrillary acid protein (GFAP) positive stellate astrocytes (inlet in **D**) and Creutzfeldt cells (inlet in **E**, black arrowhead). Demyelination **(E-H)**: H & E stained section **(E)** shows that the margin between the lesion and the surrounding neural parenchyma is relatively sharp (indicated by the dotted line between myelinated (My) and demyelinated (De) regions). Luxol fast blue histochemical preparation **(F)** confirms almost complete loss of myelin in the lesion, while immunostaining for neurofilaments **(G)** shows that the axons in the same area are well-preserved. Immunostaining for CD68 **(H)** further highlights the margin between demyelinated and myelinated areas and reveals numerous foamy macrophages within the demyelinated foci. Scale bar: 100 μm (**A-H**); 10 μm (inlets in **D** and **E**).

Given the diagnostic uncertainty – disease progression despite treatment with IVMP – needle biopsy was undertaken of the large left parietal lesion. The biopsy (Figure 2A-H) demonstrated frequent perivascular cuffs of T lymphocytes, macrophages and fewer B lymphocytes. Sheets of macrophages with foamy cytoplasm were evident in the neural parenchyma admixed with frequent GFAP positive reactive appearing astrocytes many of which showed abundant cytoplasm and peripherally placed nuclei (inlet in D). Occasional Creutzfeldt cells (inlet in E) were also identified, which is a characteristic albeit not specific feature of inflammatory demyelination. Visible on haematoxylin-eosin stained section and further highlighted on luxol fast blue special stain for myelin was the relatively sharp margin between well-myelinated areas and regions with near complete absence of myelin. Axons, however, were well-preserved throughout. Furthermore, immunostaining for mutant isocitrate dehydrogenase 1 protein harbouring R132H mutation, which is present in a large proportion of diffuse gliomas [3], was negative in the whole specimen. Hence, there was no evidence of underlying glial neoplasm. In addition, neither T lymphocytes nor B lymphocytes showed cytological atypia thereby providing no support for a neoplastic lymphoid proliferation. The Ki67 proliferation index was mildly increased in keeping with reactive proliferation of the macrophages within the lesion.

Following histological confirmation of demyelination, and six weeks after first presentation, treatment with natalizumab 300 mg IV once every four weeks was started. As part of her peri-operative care, she was also given oral dexamethasone, initially in a dose of 4 mg bd, tapered in steps of 1 mg bd every two weeks. The last dose of dexamethasone was taken one week prior to her first follow-up MRI (Figure 1M-O) four weeks after commencing natalizumab. At this time point improvement in language fluency and content was recorded, underpinned by MRI evidence of reduction in size of all lesions, and no new lesions (Figure 1M,N). After the fourth natalizumab infusion mini mental state examination score was 27/30 (pre-treatment score = 18) and Addenbrooke’s cognitive examination (ACE-R) score 82/100 (pre-treatment score = 61). The second video illustrates the improvement of the patient’s cognitive function (Additional file 2). No further relapses were observed after 16 months of clinical follow-up and 14 months after commencing natalizumab treatment (MRI Figure 1O-Q). However, two complex-partial epileptic seizures occurred six and eight months after disease onset. After starting treatment with lamotrigine 25 mg bd no further seizures occurred.

## Conclusion

Severe cognitive impairment is unusual at presentation of MS, and alternative causes need to be ruled out [2].

Gerstmann’s syndrome has been reported in highly active relapsing-remitting MS in combination with other cognitive deficits [4]. In a large series of pathologically confirmed tumefactive MS cognitive abnormalities were found to be frequent (43% in 168 patients) with higher cognitive involvement (aphasia, apraxia and agnosia) in 25% of cases. However, these cases were polysymptomatic [5]. Isolated lesions causing a specific cognitive syndrome such as in our case are exceptionally rare.

In most instances of demyelination, the associated clinical picture, MRI appearances, and CSF findings are sufficient to make a diagnosis. Despite the presence of oligo-clonal bands predominant in the CSF, however, the clinical deterioration and lack of treatment response to high-dose i.v. steroids associated with an increase in size of the tumefactive lesion and the occurrence of additional brain lesions prompted us to obtain a brain biopsy.

DWI, which provides estimates of water diffusion in biologic tissues, can be used in the assessment of tumours. Diffusion within a neoplasm is a marker of its cellularity as cells constitute a barrier to diffusion. As CNS lymphomas are highly cellular tumours, diffusion is restricted, making them appear hyper-intense on DWI and hypo-intense on apparent diffusion coefficient (ADC) maps [6]. However, large tumefactive lesions, such as in our case, may be indistinguishable from neoplasms as both can lead to mass effect/oedema, a hypo-intense rim on T_2_ weighted scans, venular enhancement, peripheral restriction on DWI, and a variable degree of ring-enhancement [7]. Clinical deterioration and rapid enlargement of the tumefactive lesion in our case, with restricted diffusion despite IVMP treatment, confounded the diagnosis of MS and underpinned the need for biopsy to exclude primary CNS lymphoma.

The findings on proton MRS of an NAA/Cr ratio well below normal may be indicative of axonal dysfunction or loss whilst the significantly increased Cho/Cr ratio suggested increased cell-membrane metabolism. However, these findings, as well as the ‘inverted doublet’ shape of the lactate curve may occur in tumefactive demyelinating lesions and tumours alike [8]. Thus, like DWI, MRS did not enable a non-invasive diagnosis in our case.

The evidence regarding prognosis and disease course of tumefactive MS remains controversial. Lesions larger than 5cm have been reported as being associated with a higher disability at follow-up [5], and patients who develop clinically definite MS after tumefactive presentation appeared to have a more aggressive disease course [9]. On the other hand, Wattamwar and co-workers described a series of 14 patients who made an excellent recovery from their large demyelinating lesions [10].

There is no standard immunomodulatory treatment for people with tumefactive MS. Treatment choices include IVMP, β-Interferons, plasma exchange (PLEX), rituximab and natalizumab [4,11-14]. In their recent review Hardy *et al*. proposed an algorithm for the acute management using IVMP and/or PLEX followed by disease modification using immunomodulatory agents [15]. The disease course in our patient suggests excellent treatment response to natalizumab, further supporting the diagnosis of relapsing MS (with tumefactive lesion leading to first presentation). Trial evidence shows natalizumab may be effective within weeks after a single infusion. In a study comparing two doses of natalizumab (3 mg and 6 mg/kilogram body weight) with placebo, Miller et al. reported significant reduction of gadolinium-enhancing lesions four weeks after their first infusion of either dose [16]. However, despite the initial lack of treatment response to IVMP in our case, a subsequent corroborating effect of peri-operative dexamethasone on the clinical course and resolution of lesions cannot be excluded. Of note, tumefactive lesions have also been reported in the context of switching immunomodulatory therapy in people with MS as described following a switch to fingolimod from natalizumab [17] and IFN [18], respectively. An inhibitory effect of fingolimod on the regulatory T cell pool has been hypothesized as a potential cause, though the evidence is so far inconclusive [19].

In summary, we present video, MRI and pathological evidence of a case of tumefactive MS which posed a diagnostic dilemma due to its atypical clinical and radiological presentation, and lack of response to steroid treatment. Clinicians, radiologists and surgeons need to appreciate the heterogeneous presentation of this disorder, and an early brain biopsy is often necessary for rapid diagnosis and management. Treatment with natalizumab may be useful in cases of tumefactive demyelination where additional evidence supports a diagnosis of relapsing MS.

### Patient consent

We confirm that a signed consent from the patient has been obtained for the publication of this report, the images and videos.

## Competing interest

KS is a PI on trials sponsored by Novartis and Roche. He has received speaking honoraria from Merck-Serono and Novartis, served on advisory boards for Biogen, Merck Inc, Merck-Serono, Novartis and Teva, and has received grant support from Novartis.

## Authors’ contributions

SG wrote the draft manuscript and prepared the videos. ZJ provided the histological analysis, prepared pathology images and was involved in drafting of the manuscript. KPB and SaG prepared MR images and were involved in drafting the manuscript. KS initiated the study, reviewed and edited the manuscript, and approved the final version. All authors read and approved the final manuscript.

## Pre-publication history

The pre-publication history for this paper can be accessed here:

http://www.biomedcentral.com/1471-2377/14/68/prepub

## Supplementary Material

Additional file 1: Video 1 Cognitive assessment of the patient before treatment. Click here for file

Additional file 2: Video 2 Cognitive assessment of the patient after three months treatment. Dysphasia, dysgraphia, finger agnosia and left-right disorientation are shown (video 1), which have subsided on subsequent assessment (video 2). Click here for file
